# Physical Literacy, According to the World Health Organization (WHO), in an Italian Preschool and Education for a Daily Movement Routine

**DOI:** 10.3390/children12010066

**Published:** 2025-01-07

**Authors:** Gaetano Raiola

**Affiliations:** Physical Education and Exercise Research Center, Pegaso Telematic University, 80143 Napoli, Italy; gaetano.raiola@unipegaso.it

**Keywords:** sedentary time, children’s health, fields of experience

## Abstract

Background and Objectives: The preschool context produces excessive sedentary behavior in children. The systematic fulfillment of structured physical activities during school time, namely a daily movement routine (DMR), can contribute to increasing the quantity of physical activity (PA) and to improving physical literacy (PL), reaching the WHO’s recommendations. Aim: The present study aims to quantify the sedentary time spent by 4- and 5-year-old preschool children and to verify the effects that a DMR could have on sedentary habits in preschool children. Method: An experimental observational study was carried out. Ad hoc questionnaires were administered to parents, teachers, and children, and an observation checklist was used to quantify the time spent in sedentary activities at school. Descriptive statistics and *t*-tests were applied for data analysis. Results: Thirty-two children attending Italian preschool participated in this study. The implementation of a DMR significantly reduced the sedentary time in preschool, decreasing the time spent sitting by 45.69% and increasing the time spent standing by 185%. Conclusions: This study confirms the hypothesis that children move for less time than recommended by the WHO and highlights the importance of making the experiences of the DMR structural, as this could be an opportunity for educational processes to enhance active lifestyles in promoting the development of physical literacy.

## 1. Introduction

Italian preschool is characterized by the organization of teaching based on “fields of experience” [1], proposing a natural non-prescriptive learning environment, which values the body and movement as instruments for interaction with the environment and for approaching knowledge [2]. For this reason, the field of experience is the basic thematic core of educational development defined by Italian schools. The “body in movement” in preschool is one of the five fields of experience through which a school offers children educational-didactic paths in which to experiment and satisfy the needs of autonomy, competence, and relationships typical of childhood. Movement play, in its spontaneous, deliberate, and organized expression [3] and, more generally, experiential teaching and learning achieved by doing and acting allows the child to reach personal, scholastic, and social autonomy appropriate to his/her age and to know and perceive his/her own body, learning to exercise its sensory-perceptive, relational, affective, expressive, and cognitive potentialities from a physical literacy perspective [4], as well as for the purposes of developing his/her own health and wellbeing. The developmental goals to be pursued consist, on the one hand, of the development of sensory-perceptive capacities and fundamental dynamic and postural motor milestones (walking, running, jumping, throwing, etc.) to adapt them to the spatial-temporal parameters in different environments. On the other hand, these goals are directed toward progressively developing the coordination of movements and mastery of one’s own behavior in interaction with the environment, which means the ability to plan and implement the most effective strategy and to realize and anticipate others’ strategies and the dynamics of objects during movement activities. Through experiences with the body and movement, children develop competence, which at this age is intended to be achieved in a global and unitary way, and contribute, to this purpose, “informal, routine and daily life activities, outdoor activities and play… the use of small tools and instruments, free or guided movement in dedicated spaces, psychomotor plays” [1], while also becoming an opportunity for health education.

The school, as a formal learning context that contributes, together with the family, to the global development of the child, has the responsibility of promoting experiences that maximize the individual educational path, bringing out the full potential of the person and promoting physical literacy in the peculiar context of the preschool classroom, which, differently from primary school [5], in many cases, has mixed-age classrooms and, therefore, presents greater problems in adequately guaranteeing the satisfaction of the movement needs of all children [6]. In addition, teachers, principals, or school administrators report barriers and/or facilitators to implementing mandated school physical activity policies [7], with the most common domains identified being ‘environmental context and resources’ (e.g., availability of equipment, time, or staff), ‘goals’ (e.g., the perceived priority of the policies in the school), ‘social influences’ (e.g., support from school boards), and ‘skills’ (e.g., teachers’ ability to implement the physical activity policies). In the current context of life, the role of the school is even more strategic due to the evident lack of environmental opportunities that are reflected in the possibilities of individual development, particularly with regard to the physical development of children, which should be adequately monitored and assessed to identify any movement difficulties early on [8].

Talking about the need for movement is “emerging” more than ever because it is not only conditioned by the (functional) needs of the body but also by the environmental, social, and cultural contingencies that characterize our existence and that stimulate less and less functionality and spontaneous adaptations of natural movements; this has important repercussions on human skills and the possibilities of interaction with the environment based on the body and movement functions. The WHO notes that the overall 24-h activity pattern is key: replacing prolonged screen time with more active play, ensuring that young children get enough good-quality sleep. Quality sedentary time spent in interactive non-screen-based activities with a caregiver, such as reading, storytelling, singing and puzzles, is very important for child development. In particular, they note that children under five need to spend less time sitting and looking at screens, sleep better and have more time to play if they are to grow up healthy, all while performing at least three hours of physical activity daily, of which at least one hour should be of a moderate-to-vigorous intensity and in which 60 min of a continuous sedentary lifestyle should not be exceeded [9].

However, the scientific literature provides evidence that the movement needs of children attending preschool [10] are not adequately satisfied, not reaching the levels recommended by the WHO [11,12] and producing negative effects on their health and wellbeing, especially considering the impact that the COVID-19 pandemic has had on people’s movement habits [13,14] and general health and wellbeing [15]. Across Europe, low compliance with physical activity recommendations in youth between countries was reported for subjectively and objectively measured physical activity [16]. In particular, there is a prevalence of sedentary behaviors, negatively influencing media availability, motorized types of commuting to and from schools, as well as inadequate PA behaviors, and European children mostly failed to meet the current recommendation about screen time [17]. In Italy, for example, the time dedicated to active play has decreased, and the time spent watching TV, playing video games, or using social media for non-educational purposes has increased; the critical issues that have emerged concern the possible association between the excessive increase in time spent on watching TV, playing video games, and using social media and greater sleep irregularities and a reduction in daily physical activity [18]. The focus on children’s lifestyles developed by the national surveillance system “OKkio alla salute” [19] which is part of the European initiative “Childhood Obesity Surveillance Initiative—COSI”, offers a detailed picture of eating habits, physical activity, sedentary lifestyle, and other aspects associated with overweight and obesity among Italian children. It emerges that Italy is one of the countries in the Mediterranean area with the highest values of excess weight in children and low levels of physical activity. In general, quantitative and qualitative data showed variability by geographical area, with evident problems in the regions of southern Italy, where the pandemic may have had more severe effects. In the Campania region (in southern Italy), there was the highest standard deviation from the national average: compared to a national average of 29.7% of the population with overweight problems and obesity (20.4% overweight, 6.9% with obesity, and 2.4% with severe obesity); in Campania itself, there is an average of 44.2% of the population with overweight problems and obesity (25.4% overweight, 12.6% with obesity and 6.2% with severe obesity). As far as the indicators of physical activity and movement are concerned, they have tended to be stable over the last 15 years (from the first survey carried out in the 2006/2007 school year to the last survey carried out in the 2022/2023 school year); this indicates that there is still much to be achieved in terms of promoting healthy lifestyles through physical activity. In 2023, 18.5% of Italian children (in Campania, 23.8%) had no physical activity the day before the survey, 41.5% still had a TV in their bedroom, and 45.1% of children spent more than 2 h a day in front of the TV/video games/tablet/cell phone. In total, 23.8% of children were inactive the day before the survey.

The national data on excess weight and insufficient physical activity in the Italian population [19] highlight many problems and concerns, as well as posing several challenges for society and institutions responsible for building strategies, policies, and interventions aimed at increasing physical activity in the entire population [20], with the help of institutional entities such as schools by promoting and using, for the sake of equity, the spaces and contexts of life in which it is possible to carry out physical activity, through the availability of a wide range of concrete opportunities to meet the different individual needs.

Considering that the context of preschool produces an excessively sedentary lifestyle in children [21], it is hypothesized that, by systematically introducing structured physical activities in the school day [22], a daily movement routine can be generated that could contribute to increase levels of physical activity, thus complying with the WHO recommendations [9]. Time management has an important pedagogical value in preschools. The rhythm of the school day is marked by well-defined moments, called “routines”. Routines are a series of moments that recur throughout the day in a constant and recurring manner, characterized by care, wellbeing, understanding, and emotional relationships. Routines provide the child with safe and constant external reference points and allow them to progressively memorize temporal concepts [23]. In routine activities, children soon feel capable and responsible and can take on the role of a tutor for peers who need help. Times dedicated to welcoming, personal hygiene, rest, school break, lunch, and free play are habitual routines. Creating a movement routine would not only have the benefit of increasing the amount of physical activity performed by children but would also develop physical literacy due to the intrinsic characteristic of the routine to harmonize knowledges, skills, motivations, and feelings connected to physical activity and movement.

The present study aimed to determine the levels of the sedentary lifestyle of children in preschool in the province of Salerno, in the Campania region; it was also proposed to verify whether the increase in the volume of physical literacy activities generated a daily habit of movement, in order to reduce sedentary behavior in the preschool context.

## 2. Materials and Methods

### 2.1. Study Design

The study was exploratory and was carried out in the context of an Italian preschool to analyze sedentary behavior in children and to verify the effectiveness of the teaching strategy of the “daily movement routine” in reducing sedentary habits in children. A survey addressed to children, parents, and teachers was conducted in order to create an exploratory study, which included systematic observations for data collection activities and a didactic-educational intervention in the experimental group to introduce physical literacy activities for 12 weeks, through the “daily movement routine”.

### 2.2. Participants

A convenience sampling method was used. The participants ware included due to their geographical location and availability to take part in the study. Thirty-two children attending two different classes (mixed-age) at the preschool in the province of Salerno in Campania Region (Italy), with their four teachers (two for each class), participated in the study. The participants were divided into two groups: an experimental and a control group, composed of 16 children aged 4 and 5 years each. The teachers had more than 10 years of experience, in an age range between 45 and 55, with a teaching diploma qualification. The limited size of the sample is linked to the exploratory nature of the study and was deemed sufficient to achieve the objectives of this investigation, which was experimental and action research and required the real composition of the classes, which in Italy has a legally binding numerical dimension; therefore, the sample was necessarily equivalent to the compositions of the two classes, and it was not possible to increase it. Before the observation and surveys began, parental consent was obtained. According to art. 9 of Regulation (EU) 679/2016, better known as GPDR (General Data Protection Regulation), ethical review and approval were waived because this was an educational research study that did not involve clinical treatment. No sensitive data were collected, meaning sensitive data “which reveal racial or ethnic origin, political opinions, religious or philosophical beliefs or trade union membership, as well as process genetic data, biometric data intended to uniquely identify a natural person, data relating to a natural person’s health or sexual life or sexual orientation”. An observational exploratory study was carried out, through systematic observation for data collection and a didactic-educational intervention aimed at introducing physical literacy activities, with the teaching strategy of the “daily movement routine”, within the already planned teaching activities.

### 2.3. Instruments

Specific assessment tools were created and used for the study purposes: an observation checklist to determine the time spent by children in sedentary activities during the school day and three “ad hoc” questionnaires to be administered to the teachers of the two classes involved, to the children, and to the children’s parents. The questionnaires were developed ad hoc due to the specific aims and type of study. Specifically, a few questions were formulated for parents to have a broader picture of children’s physical activity levels outside of school and to actively involve them in the research process. For teachers and children the questions were developed as follows:

1. The questionnaire administered to the parents consisted of 4 closed-ended questions with different answer options and aimed at knowing
  - Whether or not the child had performed physical activity during the seven days preceding the compiling of the questionnaire (choosing between 2 answer options: yes/no),
  - In the case of an affirmative answer to the first question, what the intensity of the physical activity performed was (choosing between 3 answer options: light, moderate, vigorous),
  - How much time their child had dedicated to activities of different intensity (choosing between 5 different options that quantified the time of activity),
  - How much time their child had spent in sedentary activities (choosing between 4 options that quantified the time of sedentary activity);
2. The ad hoc questionnaire for children, presented as a “physical activity pyramid game” was administered individually by teachers on a tablet through the tool “Learning app” and consisted of placing an image representing a specific physical activity (walking to school, doing organized physical activity, tidying up toys, playing outdoors, playing with the bike, going on trips/excursions, helping with household chores) or sedentary behavior (using electronic devices, TV) on an empty pyramid scheme (inspired to the pyramid image) of physical activities whose base corresponded to a minimum frequency of once a week, which increasingly intensified to the apex that corresponded to a daily frequency of at least one hour a day. This questionnaire investigated the type and quantity of physical activity of children outside of school time, and it took about 15 min to complete;
3. The ad hoc questionnaire for teachers consisted of 14 closed-ended questions with different answer options, to investigate the students’ physical activity habits during school hours as well as the teachers’ perceptions of the importance of physical activity and of their own skills in promoting children’s physical literacy;
4. An observation checklist of the time, recorded in minutes, that children spend in movement or sitting activities during the school day, in particular during the welcome, the break, the free play, and the structured teaching activities, to be filled in by the teachers, excluding the activities carried out in the afternoon hours, when children played open and independent activities or rested.

### 2.4. Procedure

The study lasted four months. It was carried out during the 2023–2024 school year and was structured in three phases.

1. In the pre-test phase, which lasted two weeks, questionnaires were administered to 24 parents, 4 teachers, and 32 children; then, observations of all the children (experimental and control group) were carried out by the teachers, specifically trained for this duty, during the school day, through five non-consecutive days within two weeks, recording on the observation checklist the time spent in dynamic or sitting activities during the school day.
2. In the test phase, which lasted three months, a movement routine was designed together with the preschool teachers. The movement routine was introduced in the experimental group and conducted daily by the teachers for 12 weeks, focusing on play related to the development of gross motor skills, with the aim of not only improving the development of these skills but above all reducing the time spent by children in sedentary behavior and building the motivation, confidence, physical competence, knowledge, and understanding to value and take responsibility for engagement in physical activities for life, which is the definition of physical literacy according to the International Physical Literacy Association (May 2014).

The activities proposed daily at least for 30 min in the routine dealt with manual dexterity, aiming and catching (i.e., throwing and/or catching the ball to a peer), and balance (walking on heels and toes, hopping on one foot, walking on bricks, maintaining the balance position). The control group, on the other hand, continued to carry out its usual activities.

3. Finally, in the post-test phase, the children were observed again by teachers during the school day, through five non-consecutive days within two weeks, recording on the observation checklist the time spent in dynamic or sitting activities during school day.

### 2.5. Data Analysis

The answers provided by the teachers, parents, and children through the questionnaires administered in the pre-test phase were described in terms of the frequency and percentages. The quantitative data resulting from the observation carried out during pre- and post-test phases and relating to the time spent in movement or sitting activities were summarized in terms of the mean and standard deviation. A *t*-test for dependent samples paired for means and a *t*-test for independent samples were performed on these data, in order to verify the pre- and post-test differences within each group and between the two groups. To assess the reliability, the internal consistency of the questionnaires was assessed through Cronbach’s α (a value of 1 indicated perfect reliability, with a cut-off of 0.70 indicating an acceptable internal consistency).

## 3. Results

**Figure 1:**
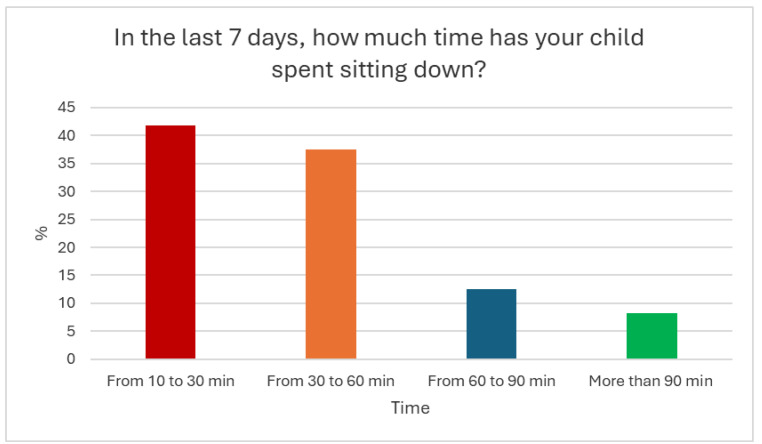
Parents’ perceptions about their child’s time spent sitting.

Twenty-four responses were obtained from the questionnaire administered to parents. The answers showed that only 29.8% of children had performed physical activity during the previous 7 days and that the type of physical activity performed mainly and for the longest time was of moderate intensity. As regards the time dedicated to sedentary activities, the majority of parents, 79.2%, perceived and reported that their child did not spend excessive time sitting, not exceeding 60 min per day (Figure 1).

The internal consistency of the questionnaires for teachers and children was acceptable in both cases (Cronbach’s α coefficient: 0.70; *p* < 0.05). The data collected through the questionnaire administered to the four teachers of the two classes involved in the study showed their perceptions relating to the movement of children during the school day, which was divided into four phases: 1. the welcome; 2. the morning teaching activity; 3. the break; 4. the afternoon activity. Despite the small number of responses by the teachers, a heterogeneity of perceptions was found (almost all the answer options were selected) with reference to the moment in which the students are most active, the planning of structured physical activities and spontaneous play in the various phases of the school day, the time spent sitting or moving during the welcome and in the afternoon, and the time spent moving during the morning teaching activity. The data on which all four teachers agreed were: (a) the time spent moving and sitting during school break and (b) the time spent sitting during teaching activities, the latter being of particular interest for the purposes of this study. In the teachers’ perception, children spent 30 to 60 consecutive minutes sitting during the morning teaching activities. The teachers also believed that movement activity was very important for children in preschool and perceived that they were adequately trained to conduct experiences in the field of experience of body and movement.

**Figure 2:**
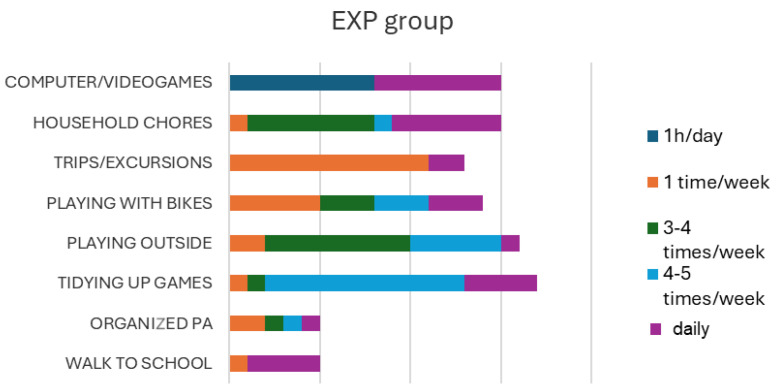
Type and frequency of physical and sedentary activities declared by children (experimental group).

**Figure 3:**
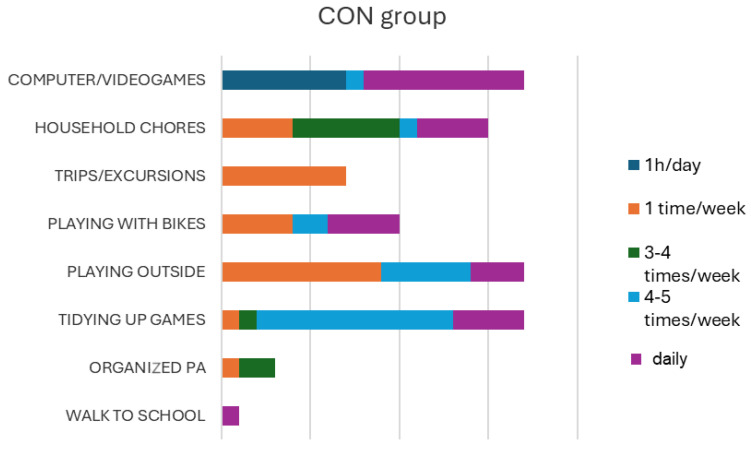
Type and frequency of physical and sedentary activities declared by children (control group).

The answers provided by children to the “pyramid of motor activities” (Figure 2 and Figure 3) showed that the activity that children carried out most frequently was sedentary (playing with computers and video games), while the activities carried out less frequently were trips/excursions and outdoor activities.

**Figure 4:**
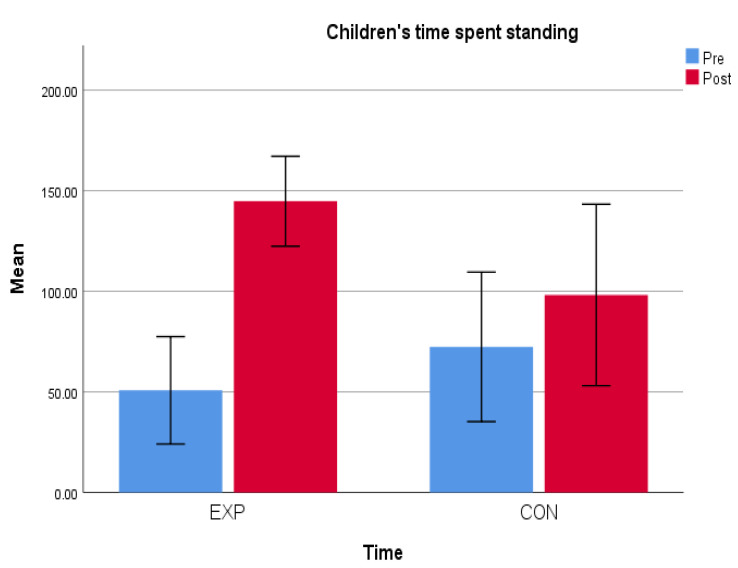
Children’s time spent standing.

**Figure 5:**
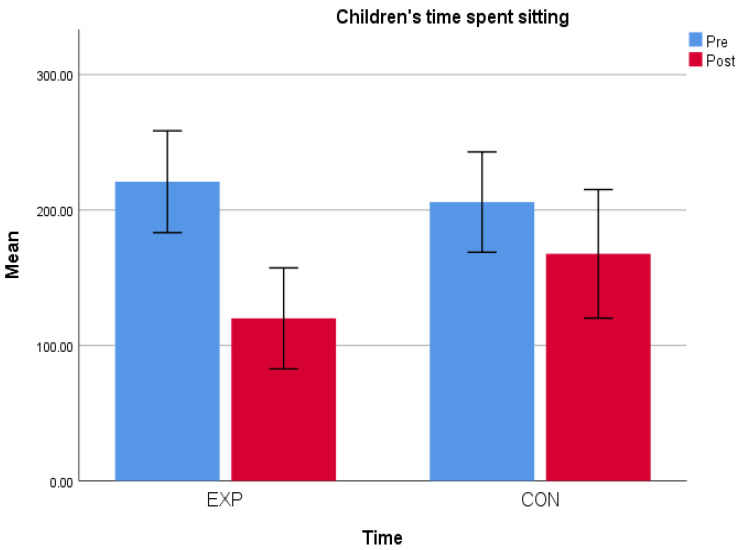
Children’s time spent sitting.

On the basis of the initial observations carried out during the pre-test phase, both groups showed high levels of sedentary time; on average, based on five observations, the experimental group was involved in sedentary activities for 215 min per day (almost 4 h), while the control group performed sedentary activities for 205 min per day (more than 3 and a half hours). From the final observations, carried out during the post-test phase, an improvement in physical activity levels and a reduction in time spent in sedentary activities were found in both groups (Figure 4 and Figure 5).

The percentage of the variation between pre and post intervention was greater in the experimental group, highlighting the benefits of the movement routine. In the comparison between the groups, there were differences both in the amount of time spent standing, which increased (Figure 4), and in the time spent sitting, which decreased (Figure 5). The average percentage of the variation in the time spent standing and sitting between the pre- and post-tests was greater in the experimental group (Table 1), confirming the validity of the movement routine implemented for the purpose of increasing the mobility of children.

The percentage of variation in the time spent standing in the experimental group was 185%, which was very high especially when compared to the 36% increase registered in the control group. The time spent sitting decreased in both groups, but in the experimental group, the percentage of variation was −45.69%, unlike the control group, which corresponded to −18% (Table 2).

The *t* test showed statistically significant differences between the two groups both for the time spent standing and for the time spent sitting (Table 3).

## 4. Discussion

The survey carried out through questionnaires administered to teachers, parents, and children defined a framework of school and life contexts in which children do not fulfil a sufficient amount of physical activity, committing instead to sedentary tasks for a lot of time, especially sedentary screen time, and little time was spent in physical activity tasks, especially in outdoor contexts.

The data emerging from this study are aligned with the damaging trends highlighted by the national surveillance system Okkio alla Salute [19] and confirm the undesirable primacy of the Campania region, which presents percentage values very far (excess or defect) from the Italian average in all the reference indicators. Particularly alarming is the confirmation, also in this study, of the high exposure of children to more than 2 h a day of TV or video games/tablet/cell phone, which turns out to be more frequent among males (64.6% vs. 55.4%) and decreases with the increase in the mother’s level of education.

It is interesting to note that the largest percentage of inactive children (33.5%) live in metropolitan/perimetropolitan areas, such as the one considered in the present study, where it is often difficult to access outdoor playground and equipment, which instead could become a crucial factor to promote physical literacy, as outdoor movement activities offer additional opportunities to promote the development of children from 3 to 6 years [24]. Furthermore, the practice of physical activities in an outdoor environment increases the perception of safety in carrying out physical activity; therefore, with adequate ongoing training on children’s movement and learning during spontaneous outdoor play and including more natural elements in the design of outdoor play areas [14], teachers could be encouraged to carry out more outdoor movement education by improving children’s awareness of the body and movement [25,26,27]. An interesting fact that emerges from the parents’ responses is that although only 29.8% of children had performed physical activity in the previous 7 days, 79.2% of parents perceived that their child did not spend excessive time sitting, not exceeding 60 min per day. These data appear contradictory; despite high levels of physical inactivity, parents consider their children’s levels of sedentary lifestyle to be “low”; this perception influences the levels of physical activity and sedentary habits of children [28] who, as emerged from this study, do not comply with the WHO recommendations.

Furthermore, in contrast to previous studies on the perception of the adequacy of their training [26,27], the teachers involved in this study declared that they were more than adequately trained to conduct experiences in the field of experience of body and movement. The perception of their (teachers) training adequacy should also correspond to the perception of physical literacy of the teachers themselves. Teachers’ physical literacy appears to be moderately associated with self-efficacy and professional competence when carrying out physical activities [29,30]. In particular, the ability to self-express and communicate and knowledge and understanding are significant predictors of teachers’ self-efficacy in classroom management and teaching strategies, as well as their professional knowledge and attitudes. Therefore, teacher training should consider these aspects of physical literacy to increase their self-efficacy and perceived competence for carrying out physical activities in school contexts and thus promote children’s physical literacy even at the preschool age [31].

Finally, from the observations carried out before and after the introduction of physical literacy activities with the teaching tool of the daily routine, it emerged that the protocol implemented in the experimental group had a positive impact on the time spent standing and sitting by children in preschool. These results suggest that an intervention aimed at promoting the physical activity and motor development of children in preschool can be effective in improving the quality of movement and promoting the development of motor skills, since the first years of childhood development are a significant period for the acquisition and application of skills that promote physical development, cognitive understanding, and social wellbeing. Implementing a daily movement routine on a permanent basis is easily applicable in Italian preschools because the school organization is autonomous and, therefore, flexible. Associating movement with a routine, like other activities that characterize the school day (such as welcoming, resting, and lunch), offers a cognitive and emotional connection to movement activities [32,33], strengthening the experience and awareness of the importance movement has in children’s lives [34]. Therefore, it is important to continue to promote educational interventions that nurture physical development from early childhood, in order to promote an active and healthy lifestyle in children and counteract increasingly early sedentary habits. It is necessary, however, to raise awareness of this problem among both parents and teachers, especially in the early years of childhood [35]. The involvement of significant adults such as parents and teachers in the study is fundamental because these figures can represent opportunities or obstacles to the dissemination of active lifestyles among children. Sensitizing adults about the risks that a sedentary lifestyle can entail helps to create a facilitating environment in which children will have greater opportunities for healthy lifestyles. To reach awareness about the problems that inactivity produces, it is necessary to start from the perceptions of the people involved to offer appropriate knowledge of the phenomenon and develop awareness with ad hoc training courses designed for adults. This training is relatively feasible with teachers who are required to do in-service training [29], while it is more problematic to involve parents who are not always available to discuss issues that they may perceive as personal or school-specific.

### Limitations and Future Directions

This study has some intrinsic limitations in selecting a low sample size, which is due to its exploratory nature, and in the use of ad hoc instruments that are related to the specific aims of the study. In particular, for this study and according to the school and teaching organization, the use of ad hoc questionnaires was the most suitable choice in terms of the time and cost-effectiveness. However, the investigation can be connected to the action research in educational contexts and classrooms, involving distinct participants: students, teachers, and other educational stakeholders, such as parents, within the system. All of these participants were engaged in activities that benefited the students, because of their role in supporting the physical literacy of children; hence, the practical applications of this study, while not generalizable, can inspire the design and implementation of daily movement routines in preschools. Future directions can consider realizing a pilot study to verify the effectiveness of the study design, instruments, and procedures used in this exploratory study, establishing their feasibility with a representative sample.

## 5. Conclusions

The aim of this study was to verify the levels of sedentary lifestyle during school time of children attending Italian preschools, providing evidence to support the benefits deriving from the implementation of a daily movement routine.

The preschool children considered in this study lived a very sedentary lifestyle according to the initial observations; however, the design and implementation of structured physical activities daily produced a double effect:

- On the one hand, they significantly reduced sedentary habits in the context of the preschool; the time spent sitting decreased by 45.69% in the experimental group compared to a reduction of 18% in the control group.
- On the other hand, they stimulated an active school time, offering greater opportunities for environmental interactions that predispose to a greater number of experiences to be lived in different fields, improving physical literacy; the time spent standing increased by 185% in the experimental group and by 36% in the control group.

This study highlights the importance of making the experiences in the field of body and movement structurally accepted into preschool organization in order to stimulate active lifestyles that contribute to the development of motor skills and physical literacy.

Physical activity promotion for preschool children should consider their natural activity patterns, which are typically spontaneous, focusing on general sensorimotor play and locomotor activities that children enjoy with fun; finally, when possible, preschool children should have access to outdoor play spaces and equipment.

Despite much scientific literature describing the determinants or correlates of physical activity among preschool children [36], there are still few studies supporting the hypothesis that improving physical activity levels among preschool children significantly improves their health [37]. In this sense, research in the exercise sciences and physical education can provide the knowledge and tools to support policy makers, governments, and local–national stakeholders in increasing physical activity levels among young people and children across Europe [38,39], counteracting the dangerous trends that are spreading [40] and verifying the correlation between physical activity levels of preschool children and their health. Sedentary behaviors should be considered alarming, especially when they are developed in the school context. The methodology explored in this study aims not simply to increase the volume of physical activities during school hours but above all to generate a daily habit of movement that develops the skills, the confidence, and the love of movement to be physically active for a lifetime.

## Figures and Tables

**Table 1:** Descriptive statistics.

	Descriptive Statistics					
	Time	Group	N	Mean	SD	Std Error Mean
**Standing time**	PRE	EXP	16	50.7604	13.3535	3.33840
		CON	16	72.3802	18.5856	4.64642
	POST	EXP	16	144.750	11.1754	2.79387
		CON	16	98.1510	22.5509	5.63773
**Sitting time**	PRE	EXP	16	220.932	18.8001	4.70004
		CON	16	205.833	18.5296	4.63242
	POST	EXP	16	119.984	18.6479	4.66198
		CON	16	167.677	23.7467	5.93670

**Table 2:** Percentage of variation in each group.

	PRE	POST	% of Variation
**Standing EXP**	50.7604	144.75	**185%**
**Standing CON**	72.3802	98.151	**36%**
**Sitting EXP**	220.932	119.984	**−45.69%**
**Sitting CON**	205.833	167.677	**−18%**

**Table 3:** *t*-test for the sitting and standing times.

	*t*-Test for Independent Samples							
		*t*	gl	Sig.	Difference in Mean	Difference in Std Error	CI 95%	
Group	Time						Lower	Upper
Standing **EXP-CON**	Pre	−3.779	30	**0.001**	−21.61979	5.72137	−33.3043	−9.93519
	Post	7.406	30	**0.000**	46.59896	6.29204	33.7489	59.44902
Sitting **EXP-CON**	Pre	2.288	30	**0.029**	15.09896	6.59922	1.62156	28.57636
	Post	−6.318	30	**0.000**	−47.69271	7.54841	−63.1086	−32.27680

## Data Availability

The raw data supporting the conclusions of this article will be made available by the author on request.
